# Maturation of Human Pluripotent Stem Cell-Derived Cerebellar Neurons in the Absence of Co-culture

**DOI:** 10.3389/fbioe.2020.00070

**Published:** 2020-02-14

**Authors:** Teresa P. Silva, Evguenia P. Bekman, Tiago G. Fernandes, Sandra H. Vaz, Carlos A. V. Rodrigues, Maria Margarida Diogo, Joaquim M. S. Cabral, Maria Carmo-Fonseca

**Affiliations:** ^1^Instituto de Medicina Molecular João Lobo Antunes, Faculdade de Medicina, Universidade de Lisboa, Lisbon, Portugal; ^2^Department of Bioengineering and Institute for Bioengineering and Biosciences, Instituto Superior Técnico, Universidade de Lisboa, Lisbon, Portugal; ^3^The Discoveries Centre for Regenerative and Precision Medicine, Universidade de Lisboa, Lisbon, Portugal; ^4^Instituto de Farmacologia e Neurociências, Faculdade de Medicina da Universidade de Lisboa, Lisbon, Portugal

**Keywords:** human induced pluripotent stem cells, cerebellar differentiation, cerebellar neurons, defined culture condition, co-culture free

## Abstract

The cerebellum plays a critical role in all vertebrates, and many neurological disorders are associated with cerebellum dysfunction. A major limitation in cerebellar research has been the lack of adequate disease models. As an alternative to animal models, cerebellar neurons differentiated from pluripotent stem cells have been used. However, previous studies only produced limited amounts of Purkinje cells. Moreover, *in vitro* generation of Purkinje cells required co-culture systems, which may introduce unknown components to the system. Here we describe a novel differentiation strategy that uses defined medium to generate Purkinje cells, granule cells, interneurons, and deep cerebellar nuclei projection neurons, that self-formed and differentiated into electrically active cells. Using a defined basal medium optimized for neuronal cell culture, we successfully promoted the differentiation of cerebellar precursors without the need for co-culturing. We anticipate that our findings may help developing better models for the study of cerebellar dysfunctions, while providing an advance toward the development of autologous replacement strategies for treating cerebellar degenerative diseases.

## Introduction

The cerebellum plays a critical role in maintaining balance and posture, coordination of voluntary movements, and motor learning in all vertebrates (for a recent review see McLachlan and Wilson, 2017). Recent evidence has indicated that this brain structure also plays a role in auditory processing tasks, reward expectation (Wagner et al., 2017), and other forms of emotional processing (Adamaszek et al., 2017). Therefore, it is not surprising that many neurological disorders are associated with abnormalities in the cerebellum (Schöls et al., 2004; Taroni and DiDonato, 2004).

A major limitation in cerebellar research has been the lack of adequate experimental models that could help identify essential molecular and cellular pathways involved in cerebellum dysfunction. Indeed, much of the current knowledge about the mechanisms of cerebellar diseases has been based on human postmortem studies, which do not inform on disease development or progression. Alternatively, animal models and immortalized human cell lines have been used (Manto and Marmolino, 2009). While these models permit in-depth investigation, they do not fully reflect the physiology and metabolism of human tissues. Thus, there is a great need for better models for the study of the human cerebellum.

The generation of cerebellar neurons using human induced pluripotent stem cells (iPSCs) aims to recapitulate early cerebellar development during human embryogenesis (Andreu et al., 2014; Butts et al., 2014; Leto et al., 2016). Pioneer studies were based on the formation of embryoid body-like structures derived from human or mouse embryonic stem cells that did differentiate into cerebellar-like cells (Su et al., 2006; Salero and Hatten, 2007; Erceg et al., 2010, 2012) but yielded a small number of immature Purkinje cells. Considering that most cerebellar disorders, namely ataxias, are associated with a loss of Purkinje cells, this technical limitation compromises the use of such embryoid bodies as a model for studying cerebellar biology and pathology.

Recent studies have generated bona fide Purkinje cells from mouse embryonic stem cells (Muguruma et al., 2010), human pluripotent stem cells (Muguruma et al., 2015), or spinocerebellar ataxia patient-derived iPSCs (Ishida et al., 2016). The maturation into functional Purkinje neurons has so far been achieved by co-culturing cells with either cerebellar granule cell precursors isolated from mouse embryos (Muguruma et al., 2015; Ishida et al., 2016) or fetal cerebellar slices (Wang et al., 2015). A co-culture system using postnatal cerebellum has produced cells with morphological and synaptogenesis features similar to mature Purkinje cells (Tao et al., 2010). However, significant variability in the efficiency to obtain functional Purkinje cells using different feeder cell sources was later reported by Wang et al. (2015). For instance, feeder-free and co-culturing with rat granular progenitors failed to sustain Purkinje cell maturation and survival, while co-culture with rat cerebellar slices did yield Purkinje cells that nevertheless were devoid of any action potential or spontaneous post-synaptic currents. In contrast, co-culture with human fetal cerebellar slices resulted in electrophysiologically active Purkinje neurons (Wang et al., 2015). Despite these important developments, the use of co-culture systems has limitations because feeder cells introduce inherent variability to the procedure, which may in turn affect its reproducibility and experimental outcomes (Akopian et al., 2010; Wang et al., 2015). Moreover, co-culture systems must be avoided when generating iPSC-derived cerebellar neurons for autologous transplantation. Thus, establishing long-term culture systems that sustain maturation of human cerebellar neurons without co-culturing is highly needed.

Here we present a novel strategy to generate distinct types of cerebellar cells that self-formed and differentiated into electrically active neurons in a defined medium. The possibility of efficiently generating cerebellar neurons from patient-derived iPSCs will facilitate drug screening and the study of specific pathways involved in disease development and progression.

## Materials and Methods

### Maintenance of Human iPSCs

We used three distinct human induced pluripotent stem cell (iPSC) lines. A cell line, termed F002.1A.13, was derived from a healthy female donor using a standard protocol (Takahashi et al., 2007). Karyotyping of these cells revealed no abnormalities, and upon subcutaneous injection into immune-deficient mice they induced the formation of tumoral masses reminiscent of teratomas (Hentze et al., 2009), thus confirming their *in vivo* differentiation potential (Supplementary Figure S1). Besides the F002.1A.13, the Gibco^®^ Human Episomal iPSC line (iPSC6.2, Burridge et al., 2011) and iPS-DF6-9-9T.B, provided by WiCell Bank, were also used. All iPSCs were cultured on Matrigel^TM^ (Corning)-coated plates with mTeSR^TM^1 Medium (StemCell Technologies). Medium was changed daily. Cells were passaged every 3–4 days (when the colonies covered approximately 85% of the surface area of the culture dish) using 0.5 mM EDTA dissociation buffer (Life Technologies). Before each differentiation process, frozen cells were thawed and cultured for 2–3 passages.

### Teratoma Assay

Animal experimentation at Instituto de Medicina Molecular was conducted strictly within the rules of the Portuguese official veterinary directorate, which complies with the European Guideline 86/609/EC concerning laboratory animal welfare, according to a protocol approved by the Institute’s Animal Ethics Committee. To assess the capacity of the F002.1A.13 cells to form teratomas, cells were collected using 0.5 mM EDTA dissociation buffer and 2 × 10^6^ cells were resuspended in mTeSR^TM^1/Matrigel 1:1 and subcutaneously injected into the flanks of 8-week-old immunocompromised mice (NGS). Animals were sacrificed with anesthetic overdose and necropsy was performed. Subcutaneous tumor and ipsilateral inguinal lymph node were harvested, fixed in 10% neutral-buffered formalin, embedded in paraffin, and 3 μm sections were stained with hematoxylin and eosin (Supplementary Figure S1A). Tissue sections were examined by a pathologist blinded to experimental groups in a Leica DM2500 microscope coupled to a Leica MC170 HD microscope camera.

### Karyotyping and Flow Cytometry of Human iPSCs

F002.1A.13 cells were incubated with colcemid (10 μg/ml; Life Technologies) for 4 h to arrest cells in metaphase. Next, cells were collected and incubated with hypotonic potassium chloride solution for 15 min at 37°C. Finally, cells were resuspended and fixed in glacial acetic acid and methanol (1:3). Karyotype analysis was performed by Genomed SA (Lisbon, Portugal) (Supplementary Figure S1B). Flow cytometry analysis for five different pluripotency markers was performed on day zero of differentiation (Supplementary Figure S1C).

### 3D Culture of Cerebellar Progenitors

To promote human iPSC aggregation into embryoid body-like floating structures, the three iPSC lines used in this study were incubated with ROCK inhibitor (ROCKi, Y-27632, 10 μM, StemCell Technologies) for 1 h at 37°C and then treated with accutase (Sigma) for 5 min at 37°C. After dissociation, cells were quickly re-aggregated using microwell plates (AggreWell^TM^800, StemCell Technologies) according to the manufacturer’s instructions. Cells were plated at a density of 1.8 × 10^6^ cells/well (6,000 cells/microwell) in 1.5 mL/well of mTeSR^TM^1 supplemented with 10 μM ROCKi. Twenty-four hours later the entire medium was replaced and cells were maintained in mTeSR^TM^1 without ROCKi for another 24 h.

Day 0 was when the aggregate culture was started. The basal differentiation medium used during days 2–21 was growth-factor-free chemically defined medium (gfCDM) (Muguruma et al., 2015), consisting of Isocove’s modified Dulbecco’s medium (Life Technologies)/Ham’s F-12 (Life Technologies) 1:1, chemically defined lipid concentrate (1% v/v, Life Technologies), monothioglycerol (450 μM, Sigma), apo-transferrin (15 μg/ml, Sigma), crystallization-purified BSA (5 mg/ml, >99%, Sigma), and 50 U/ml penicillin/50 μg/ml streptomycin (PS, Life Technologies). The medium was also supplemented with insulin (7 μg/ml, Sigma).

**FIGURE 1:**
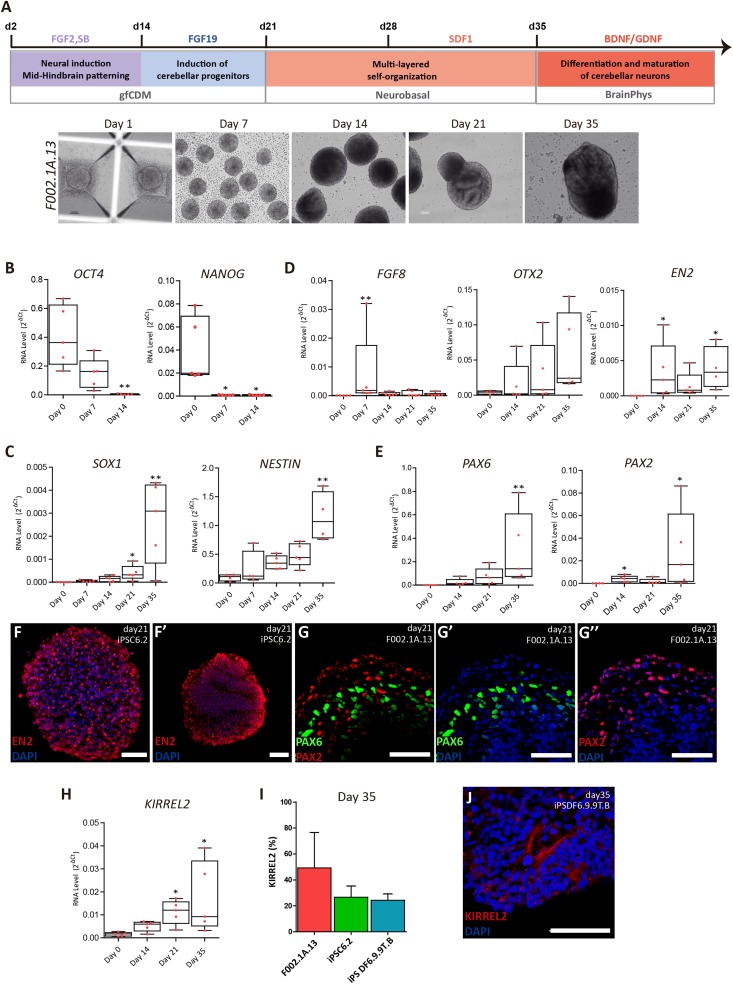
Differentiation of cerebellar progenitors in 3D culture. **(A)** Schematics illustrating the 3D culture conditions used to induce differentiation of iPSCs to cerebellar neurons. Representative bright field images of cell aggregates taken at the indicated time points. Scale bar, 100 μm. **(B–E)** qRT-PCR analysis of cultures derived from F002.1A.13 cells for the indicated mRNAs. The graphs depict mRNA expression levels (2^–ΔCt^) relative to GAPDH. Each dot represents data from an independent experiment (*n* = 5). One-way ANOVA (Dunn’s Multiple Comparison Test), ^∗^*p* < 0.05, ^∗∗^*p* < 0.01; error bars represent SEM. **(F,G)** Immunofluorescence of cultures derived from the indicated iPSC lines for EN2 **(F,F’)**, PAX6 **(G,G’),** and PAX2 **(G,G”)** on day 21 of differentiation (scale bar, 50 μm). **(H)** qRT-PCR analysis of cultures derived from F002.1A.13 cells for mRNA encoding the KIRREL2 protein. The graph depicts mRNA expression levels (2^–ΔCt^) relative to GAPDH. Each dot represents data from an independent experiment (*n* = 5). One-way ANOVA (Dunn’s Multiple Comparison Test), ^∗^*p* < 0.05; error bars represent SEM. **(I)** Flow cytometry analysis of KIRREL2^+^ cells on day 35. The graph depicts the proportion of KIRREL2^+^ cells in cultures derived from the indicated iPSC lines. Two independent experiments were performed for each cell line; error bars represent SEM. **(J)** Immunofluorescence for KIRREL2 in a culture derived from DF6.9.9T.B cells on day 35 (scale bar, 50 μm).

Recombinant human basic FGF (FGF2, 50 ng/ml, PeproTech) and SB431542 (SB, 10 μM, Sigma) were added to culture on day 2. The entire medium was replaced by gfCDM (supplemented with insulin, FGF2 and SB) on day 5. On day 7, the floating aggregates were transferred from microwell plates to ultra-low attachment 6-well plates (Costar, Corning) and cultured at a density of 1 × 10^6^ cells/mL in 1.8 mL/well. Medium was replaced and 2/3 of the initial amount of FGF2 and SB was added. Recombinant human FGF19 was added to culture on day 14, and the entire medium was replaced by gfCDM (supplemented with insulin and FGF19) on day 18. From day 21 onward, the aggregates were cultured in Neurobasal medium (Life Technologies) supplemented with GlutaMax I (Life Technologies), N2 supplement (Life Technologies), and PS. The entire medium was then replaced weekly. Recombinant human SDF1 (300 ng/ml, PeproTech) was added to culture on day 28 (Figure 1A).

### Maturation of Cerebellar Neurons in 2D Culture

On day 35 of differentiation, aggregates were dissociated using accutase (Sigma) and cells were plated on wells coated with poly-L-ornithine (15 μg/mL, Sigma) and Laminin (20 μg/mL, Sigma), at a seeding density between 80,000 cells/cm^2^. Cells were cultured in BrainPhys^TM^ Neuronal Medium (STEMCELL Technologies), supplemented with NeuroCult^TM^ SM1 Neuronal Supplement (STEMCELL Technologies), N2 Supplement-A (StemCell Technologies), recombinant Human Brain Derived Neurotrophic Factor (BDNF, PeproTech, 20 ng/mL), recombinant Human Glial-Derived Neurotrophic Factor (GDNF, PeproTech, 20 ng/mL), dibutyryl cAMP (1 mM, Sigma), and ascorbic acid (200 nM, Sigma). One-third of the total volume was replaced at every 2–3 days.

### Aggregate Size Analysis

To monitor the size of aggregates, images were acquired at different time-points using a Leica DMI 3000B microscope with a Nikon DXM 1200F digital camera. The aggregate diameter was estimated using the Mathworks computer tool (MATLAB), as described (Miranda et al., 2015).

### Immunostaining

Aggregates were fixed in 4% paraformaldehyde (PFA, Sigma) for 20 min at 4°C followed by washing in Phosphate buffered saline (PBS, 0.1M) and overnight incubation in 15% sucrose at 4°C. Next, aggregates were embedded in 7.5% v/v gelatin/15% v/v sucrose and frozen in isopenthane at −80°C. Sections with approximately 12 μm in thickness were cut on a cryostat-microtome (Leica CM3050S, Leica Microsystems), collected on Superfrost^TM^ Microscope Slides (Thermo Scientific), and stored at −20°C. Finally, sections were de-gelatinized for 45 min in PBS at 37°C before being processed for immunohistochemistry.

Immunostaining was performed on either sections of 3D aggregates or on cells plated on coverslips (2D cultures). Cells in 2D cultures were fixed in 4% PFA for 20 min at 4°C. Samples were then incubated in 0.1 M Glycine (Millipore) for 10 min at room temperature (RT), permeabilized with 0.1% Triton X-100 (Sigma) for 10 min at RT, and blocked with 10% v/v fetal goat serum (FGS, Life Technologies) in TBST (20 mM Tris-HCl pH 8.0, 150 mM NaCl, 0.05% v/v Tween-20, Sigma) for 30 min at RT. Next, samples were incubated overnight at 4°C with the primary antibodies (Supplementary Table S1) diluted in blocking solution. Secondary antibodies (goat anti-mouse or goat anti-rabbit IgG, Alexa Fluor^®^–488 or –546, 1:500 v/v dilution, Molecular Probes) were incubated for 30 min at RT. Nuclei were stained with 4′,6-diamidino-2-phenylindole (DAPI, 1.5 μg/mL; Sigma). For phalloidin staining, cells were incubated with Alexa Fluor^®^ 488 Phalloidin (1:40 in PBS, Life Technologies). Finally, samples were mounted in Mowiol (Sigma). Fluorescence images were acquired with Zeiss LSM 710 or Zeiss LSM 880 Confocal Laser Point-Scanning Microscopes.

### Quantitative Real Time (qRT)-PCR

Total RNA was extracted using High Pure RNA Isolation Kit (Roche), according the instructions provided by the manufacturer. Total RNA was converted into complementary cDNA with Transcriptor High Fidelity cDNA Synthesis Kit (Roche) using 500 ng of RNA. RNA levels were measured using 10 ng of cDNA and 250 μM of each primer. Taqman^®^ Gene Expression Assays (20X, Applied Biosystems) were selected for mRNAs from the following genes: *NANOG* (HS02387400-g1), *OCT4* (HS00999634-sh), *PAX6* (HS00240871-m1), *SOX1* (HS01057642-s1), *EN2* (Hs00171321_m1), *KIRREL2* (Hs00375638_m1), and *GAPDH* (HS02758991-g1). SYBR^®^ green chemistry was used to analyze mRNAs from the following genes: *ALDOC, ATOH1, BARHL1, CBLN1, CORL2, EN2, FGF8, GAD65, GRID2, L7/PCP2, LHX5, NESTIN, NEUROGRANIN, OLIG2, OTX2, PARVALBUMIN, PAX2, TBR1, TBR2* and *VGLUT1* (Supplementary Table S2). All PCR reactions were done in duplicate or triplicate, using the StepOne^TM^ or ViiA^TM^ 7 RT-PCR Systems (Applied BioSystems). Quantification was performed by calculating the ΔCt value using GAPDH as a reference and results are shown as mRNA expression levels (2^–ΔCt^) relative to GAPDH.

### Flow Cytometry

Aggregates were dissociated to single cells with accutase for 7 min at 37°C. Adherent cells in 2D cultures were detached by incubation with accutase at 37°C for 5 min. The enzyme was inactivated by addition of serum-containing medium. After centrifugation, the cell pellet was washed with PBS, fixed in 2% v/v PFA, and stored at 4°C. For Ki67 analysis, cells were fixed drop by drop with 70% v/v ethanol (previously stored at −20°C) and stored at −20°C. For cell staining, eppendorf tubes were coated with 1% v/v bovine serum albumin (BSA; Life Technologies) solution in PBS for 15 min. Samples stored in 2% v/v PFA were placed in the coated eppendorf tubes and centrifuged at 1000 rpm for 5 min. Samples stored in 70% v/v ethanol were also placed in coated eppendorf tubes and centrifuged at 1000 rpm for 10 min. Then, cells were washed twice with PBS. Surface staining: for each experiment, approximately 500,000 cells were resuspended in primary antibody diluted in 3% v/v BSA solution in PBS and incubated for 30 min at RT. Then, cells were washed with PBS, resuspended in 3% v/v BSA solution in PBS and incubated with secondary antibodies for 15 min at RT. Finally, cells were washed twice with PBS, resuspended in PBS and analyzed in a FACSCalibur^TM^ flow cytometer (Becton Dickinson). Intracellular staining: for each experiment, approximately 500,000 cells were resuspended in 3% v/v normal goat serum (NGS, Sigma). The cell suspension was centrifuged at 1000 rpm for 3 min. Next, cells were permeabilized with 1% saponin (Sigma) diluted in a solution of 3% NGS in PBS for 15 min at RT. After washing three times with 1% NGS, cells were resuspended in primary antibody solution (in 3% NGS) and incubated for 1h at RT (Supplementary Table S3). Cells were then washed three times with 1% NGS, and incubated for 45 min in the dark with the secondary antibody (in 3% NGS). Secondary antibodies included goat anti-mouse and anti-rabbit IgG Alexa Fluor – 488 (Invitrogen, 1:500), and anti-mouse IgG-PE (1:500, Miltenyi Biotec). After washing, cells were resuspended in PBS and analyzed in a FACSCalibur^TM^ flow cytometer (Becton Dickinson). As a negative control, cells were incubated with secondary antibody only. For each experimental sample, 10 000 events were collected within the defined gate, based on side scatter (SSC) and forward scatter (FSC). Results were analyzed using FlowJo.

### Single Cell Calcium Imaging

For single cell calcium imaging (SCCI), aggregates were dissociated using accutase and cells were plated on glass bottom micro-well chambers previously coated with poly-D-lysine (MatTek) and Laminin (20 μg/mL, Sigma), at a seeding density of 80 000 cells/cm^2^. At different time-points of differentiation, cells were loaded with 5 μM Fura-2 AM (Invitrogen) in Krebs solution (132 mM NaCl, 4 mM KCl, 1.4 mM MgCl2, 2.5 mM CaCl_2_, 6 mM glucose, 10mM HEPES, pH 7.4) for 45 min at 37°C in an incubator with 5% CO_2_ and 95% atmospheric air. Dishes were washed in Krebs solution and observed with an inverted microscope with epifluorescence optics (Axiovert 135TV, Zeiss). Cells were continuously perfused with Krebs solution and stimulated by applying high-potassium Krebs solution (containing 10–100 mM KCl, isosmotic substitution with NaCl), 2 μM ionomycin, or 100 μM histamine. Ratio images were obtained from image pairs acquired every 200 ms by exciting the cells at 340 and 380 nm. Excitation wavelengths were changed through a high-speed switcher (Lambda DG4, Sutter Instrument, Novato, CA, United States). The emission fluorescence was recorded at 510 nm by a cooled CDD camera (Photometrics CoolSNAP fx). Images were processed and analyzed using the software MetaFluor (Universal Imaging, West Chester, PA, United States). Regions of interest were defined manually.

### Dendritic Spine Classification

After phalloidin-staining, dendritic spines were imaged with either a Zeiss LSM 710 or a Zeiss LSM 880 Confocal Laser Point-Scanning Microscope, using the 63x Plan-Apochromat oil objective. Regions with low density of neurons were selected to allow visualization of individual dendrites and spines. Three representative images were analyzed per experiment. For each dendritic spine, the head width and the neck length were measured manually using ImageJ software.

### Patch-Clamp Recordings

Whole-cell patch-clamp recordings were obtained from cerebellar neurons visualized with an upright microscope (Zeiss Axioskop 2FS) equipped with differential interference contrast optics using a Zeiss AxioCam MRm camera and an x40 IR-Achroplan objective. During recordings, cells were continuously superfused with artificial cerebrospinal fluid (aCSF) containing 124 mM NaCl, 3 mM KCl, 1.2 mM NaH2PO_4_, 25 mM NaHCO_3_, 2 mM CaCl_2_, 1 mM MgSO_4_, and 10 mM glucose, which was continuously gassed with 95%O_2_/5% CO_2_. Recordings were performed at room temperature in current-clamp or voltage-clamp mode [holding potential (Vh) = -70 mV] with an Axopatch 200B (Axon Instruments) amplifier, as performed elsewhere (Felix-Oliveira et al., 2014). In the current-clamp mode, the step-and-hold stimulation protocol included 11 steps of 500 ms long depolarization pulses. The first injection current was −25 pA and the subsequent ones increased progressively until 225 pA. Synaptic currents and action potential activity were recorded using patch pipettes with 4–7 MΩ resistance filled with an internal solution containing 125 mM K-gluconate, 11 mM KCl, 0.1 mM CaCl_2_, 2 mM MgCl_2_, 1 mM EGTA, 10 mM HEPES, 2 mM MgATP, 0.3 mM NaGTP, and 10 mM phosphocreatine, pH 7.3, adjusted with 1 M NaOH, 280-290 mOsm. Acquired signals were filtered using an in-built, 2-kHz, three-pole Bessel filter, and data were digitized at 5 kHz under control of the pCLAMP 10 software program. The junction potential was not compensated for, and offset potentials were nulled before gigaseal formation. The resting membrane potential was measured immediately upon establishing whole-cell configuration. In the voltage-clamp mode, spontaneous miniature postsynaptic currents were recorded in aCSF solution for 5 min. After this period, the sCSF solution was supplemented with 500 nM TTX (tetrodotoxin, a voltage-dependent sodium channel blocker), 5 μM CNQX (6-cano-7-nitroquinoxaline-2, 3-dione, a glutamate AMPA receptor antagonist), and 50 μM DL-APV (DL-(-)-2-amino-5-phosphonopentanoic acid, a glutamate NMDA receptor antagonist), for the specific recording of miniature post-synaptic inhibitory currents, miPSCs (Rombo et al., 2016). To silence miPSCs and confirm their GABAergic nature, 10 μM bicuculline (a GABA_A_ receptor antagonist) was used at the end of the recording. The same protocol was performed but first inhibiting miPSCs and then Glutamatergic responses at the end of the recording. Analysis was performed offline using the spontaneous event detection parameters of the Synaptosoft Minianalysis software, the amplitude threshold for event detection being set at 3x the average root-mean-square noise.

## Results

### Induction of Cerebellar Differentiation in a 3D Culture System

Three distinct lines of human iPSCs (F002.1A.13; iPSC6.2; and iPSDF6.9.9T.B) were induced to differentiate into cerebellar neurons. A total of 10 independent differentiation experiments were carried out using the F002.1A.13 cells, 8 using the iPSC6.2 cells, and 4 using the iPSDF6.9.9T.B cells. Initially, cells self-assembled spontaneously in suspension culture in mTESR1 medium. On day 2, the medium was replaced by gfCDM, on day 21 by neurobasal medium, and on day 35 by BrainPhys medium (Figure 1A). On day 2, fibroblast growth factor 2 (FGF2), insulin, and transforming growth factor β-receptor blocker SB431542 were added. Fibroblast growth factor 19 (FGF19) was then added between days 14 and 21, followed by stromal cell-derived factor 1 (SDF1) between days 28 and 35, as previously described (Muguruma et al., 2015). To maximize neural commitment, the sizes of 3D cell aggregates were controlled using V-shaped microwell plates. On day zero of differentiation, approximately 6,000 cells were plated per microwell and aggregation was induced by centrifugation. On day 7, the floating aggregates were transferred to ultra-low attachment 6-well culture plates. As shown in Figure 1A and Supplementary Figure S2A, on day 7 the three iPSC lines formed 3D aggregates that were homogeneous in size and shape. After day 7, the aggregates started to grow and vary in size and morphology (Figure 1A and Supplementary Figures S2A,B).

As expected, on day zero of differentiation the iPSCs expressed the pluripotency and self-renewal genes *OCT4* and *NANOG* (Figure 1B). On day 7, *OCT4* and *NANOG* mRNA levels were significantly reduced and on day 14 they were almost undetectable, indicating that cells were committed to differentiate. Starting on day 7 and going onward until day 35, aggregates progressively expressed higher mRNA levels encoding the neural stem cell markers SOX1 and NESTIN (Figure 1C). Immunofluorescence analysis further revealed that most cells within the aggregates expressed NESTIN and PAX6 (Supplementary Figure S2C). Altogether, these results support the iPSC-derived neural commitment.

As shown in Figure 1D, the level of RNA transcribed from *FGF8*, a gene encoding a signaling protein required for early cerebellum development (Chi et al., 2003), was at its highest on day 7 of differentiation and dropped thereafter. Figure 1D also shows that *OTX2* mRNA levels were higher on day 35. *OTX2* is a homeobox gene required for cerebellum development that is inhibited by FGF8 (Frantz et al., 1994; Larsen et al., 2010). As expected, *OTX2* levels increased only after *FGF8* mRNA levels dropped. On days 14 through 35, we also detected the expression of *EN2* (Figure 1D), another homeobox gene required to generate a fully functional cerebellum (Zec et al., 1997). These findings indicate that our iPSC-derived 3D aggregates expressed critical genes required for early cerebellum morphogenesis.

We next focused on detecting specific cerebellar progenitors. Starting on day 14, we detected increasing levels of *PAX6* mRNA (Figure 1E), which encodes a transcription factor required for the development of all cerebellar glutamatergic neurons (Yeung et al., 2016). We further found significant expression of *PAX2* (Figure 1E), which is a gene that encodes a transcription factor involved in normal functioning of mid-hindbrain junction and further cerebellar commitment (Urbánek et al., 1997). The expression of proteins encoded by the *EN2*, *PAX6*, and *PAX2* genes was also detected by immunostaining (Figures 1F’,G’,G”), confirming the efficient mid-hindbrain patterning and further cerebellar commitment within the aggregates.

As Figure 1H shows, on day 35 we detected a significant expression of *KIRREL2* mRNA, which encodes a cell adhesion molecule found on the surface of cerebellar GABAergic progenitors, including Purkinje cell precursors (Mizuhara et al., 2010). The results of flow cytometry analysis show that the proportion of KIRREL2 positive cells on day 35 was approximately 50% in F002.1A.13-derived cell aggregates, and about 30% in iPSC6.2 and iPSDF6.9.9T.B-derived cell aggregates (Figure 1I). KIRREL2 protein expression was further confirmed by immunofluorescence (Figure 1J and Supplementary Figure S2D), indicating that after 35 days of differentiation aggregates derived from the three iPSC lines had cerebellar GABAergic progenitors.

### Cerebellar Progenitors in 3D Culture Self-Organize Into Polarized Neuroepithelium

**FIGURE 2:**
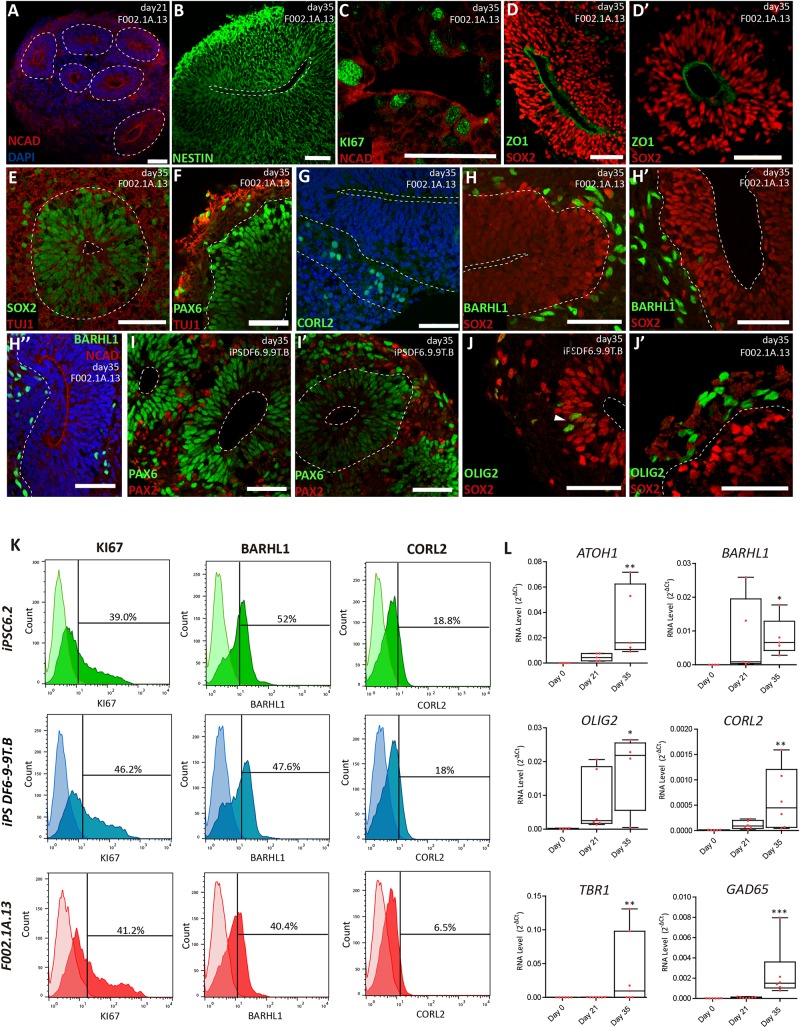
Cerebellar progenitors self-organize into structures reminiscent of the neural tube. **(A–J)** Immunofluorescence analysis on days 21 and 35 of differentiation of the indicated iPSC lines. Blue staining corresponds to nuclei labeled with DAPI. **(A)** Dashed lines delineate neural rosettes containing a lumen-like center marked by N-Cadherin (NCAD). **(B)** Neuro-epithelial rosette stained for Nestin. **(C)** Proliferating cells positive for KI67 located close to the lumen marked by NCAD. **(D,D’)** Apical layer of SOX2 positive cells surrounding the lumen marked by ZO1, a tight junction protein. **(E)** Dashed lines delineate the lumen and the boundary between the apical layer of SOX2 positive cells and the basal layer containing cells expressing neuron-specific class III beta-tubulin (TUJ1). **(F)** Dashed line delineates the boundary between the apical layer of PAX6 positive cells and the basal layer containing cells expressing TUJ1. **(G)** Dashed lines delineate the lumen and the boundary between the apical and basal layers; cells stained green express CORL2, a marker of Purkinje cell precursors. **(H,H’)** Dashed lines delineate the lumen and the boundary between the apical layer of SOX2 positive cells and the basal layer containing cells expressing BARHL1, a marker for granule cell precursors. **(H”)** Dashed line delineates the boundary between the apical layer (lumen marked by NCAD) and the basal layer containing cells expressing BARHL1. **(I,I’)** Dashed lines delineate the lumen and the boundary between the apical layer of PAX6 positive cells and the basal layer containing cells expressing PAX2, a marker for GABAergic interneurons. **(J)** Dashed line delineates the lumen; arrowhead indicates cells expressing OLIG2 (a marker for newly formed Purkinje cells) interspersed among SOX2 positive cells. **(J’)** Dashed line delineates the boundary between the apical layer of SOX2 positive cells and the basal layer containing cells expressing OLIG2. Scale bars, 50 μm. **(K)** Flow cytometry analysis of cells expressing KI67, BARHL1 and CORL2 on day 35 of differentiation of the indicated iPSC lines (*n* = 3 per marker). **(L)** qRT-PCR analysis of cultures derived from F002.1A.13 cells at the indicated time points. The graphs depict mRNA expression levels (2^–ΔCt^) relative to GAPDH. Each dot represents data from an independent experiment (*n* = 5). One-way ANOVA (Dunn’s Multiple Comparison Test), ^∗^*p* < 0.05, ^∗∗^*p* < 0.01, ^∗∗∗^*p* < 0.001; error bars represent SEM.

On day 21, aggregates derived from the three iPSC lines displayed characteristic hollow structures with a radial organization (neural rosettes) reminiscent of the neural tube. These structures showed apical-basal polarity, with the apical side marked by a strong signal of *N*-cadherin (NCAD, Figure 2A). Polarized neuroepithelial structures were more prominent on day 35 (Figures 2B–J). Dividing cells stained by the proliferation marker KI67 were enriched on the apical (luminal) side of the neuroepithelium (Figure 2C and Supplementary Figure S3A). Immunostaining for SOX2 (Figures 2D,D’,E) and PAX6 (Figure 2F) further showed that the proliferating apical cell layer was mainly composed of cerebellar progenitors. In contrast, cells expressing neuron-specific class III beta-tubulin (TUJ1) formed a separate, more basally located layer (Figures 2E,F and Supplementary Figure S3C). Cells located in the basal compartment expressed markers for precursors of Purkinje and granule cells, respectively CORL2 (Figure 2G) and BARHL1 (Figures 2H,H’,H” and Supplementary Figure S3D), and the marker for maturing GABAergic neurons GAD65 (Supplementary Figure S3E). Basally located cells further expressed PAX2 (Figures 2I,I’ and Supplementary Figure S3F), which is a marker of GABAergic interneurons and their precursors in the developing cerebellum (Zhang and Goldman, 1996; Maricich and Herrup, 1999). Cells positive for OLIG2, a transcription factor expressed in neurogenic progenitors and nascent Purkinje cells (Seto et al., 2014; Ju et al., 2016), were also predominantly located in the basal layer (Figures 2J,J’ and Supplementary Figures S3G,G’).

The proportion of different cell populations within the aggregates was quantified by flow cytometry (Figure 2K). At day 35, 3D aggregates were composed by 42.1 ± 2.1% of KI67^+^ proliferating cells, 46.7 ± 3.4% of BARHL1^+^ granule cell precursors, and 14.4 ± 3.9% of CORL2^+^ Purkinje cell precursors (Means ± SEM of the three iPSC lines). Significant levels of mRNAs encoding specific markers for different types of cerebellar neurons were also detected by qRT-PCR on day 35 (Figure 2L). These markers included, in addition to BARHL1, OLIG2, CORL2, and GAD65, the transcription factor ATOH1 (required for differentiation of cerebellar granule neurons) and TBR1 (expressed in deep cerebellar nuclei).

### Maturation of Cerebellar Neurons in 2D Culture

**FIGURE 3:**
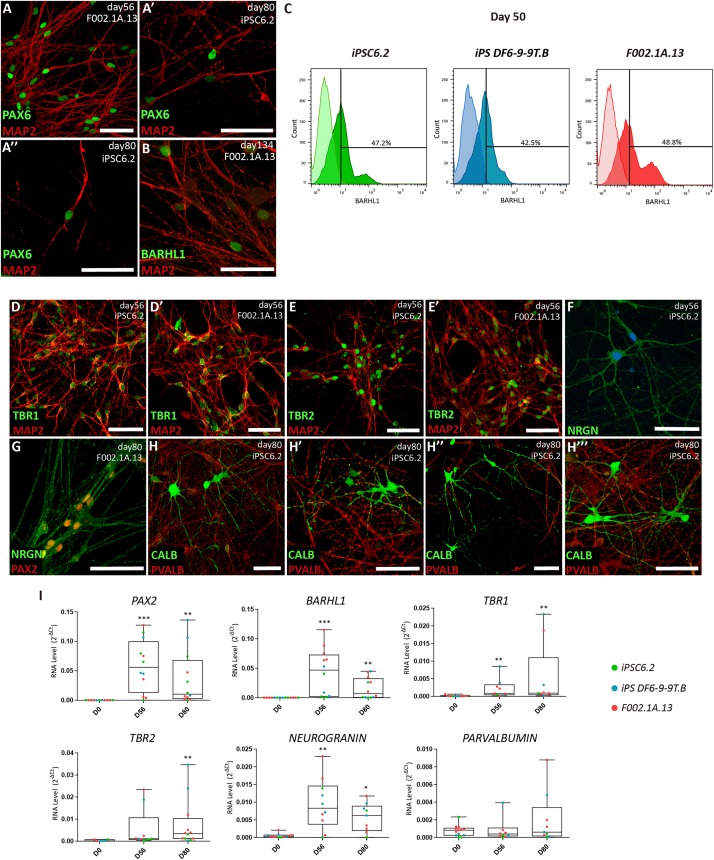
Maturation of distinct types of cerebellar neurons in 2D culture. **(A,B)** Immunofluorescence analysis on days 56, 80, and 134 of differentiation of the indicated iPSC lines. The depicted MAP2^+^ neurons have the nuclei stained for PAX6 and BARHL1. Scale bars, 50 μm. **(C)** Flow cytometry analysis of BARHL1^+^ cells on day 50 of differentiation of the indicated iPSC lines. **(D–H)** Immunofluorescence analysis on days 56 and 80 of differentiation of iPSC lines using the indicated antibodies. In panel F, nuclei are stained with DAPI (blue). Scale bars, 50 μm. **(A’, A”, E’, H’–H”’)** are used for different captures using the same antibodies combinations, regardless of the iPSC line and differentiation day. **(I)** qRT-PCR analysis of cultures derived from each iPSC line at the indicated time-points. Box-plot diagrams depict mRNA expression levels (2^–ΔCt^) relative to GAPDH. Each color-coded dot represents data from one differentiation experiment. One-way ANOVA (Dunn’s Multiple Comparison Test), ^∗^*p* < 0.05, ^∗∗^*p* < 0.01, ^∗∗∗^*p* < 0.001; error bars represent SEM.

To promote further maturation of cerebellar neurons, aggregates were dissociated on day 35. Cells were then transferred to laminin-coated plates and cultured in serum-free BrainPhys^TM^ medium (Bardy et al., 2015) supplemented with BDNF and GDNF (Figure 1A). After 15 days in 2D culture, i.e., on day 50 and onward, cells were analyzed by immunofluorescence, flow cytometry and qRT-PCR (Figure 3). Immunofluorescence was performed using antibodies to microtubule-associated protein 2 (MAP2), a neuron-specific protein that stabilizes microtubules in the dendrites of postmitotic neurons. The results revealed MAP2^+^ neurons with the nuclei stained for PAX6 and BARHL1 (Figures 3A,B and Supplementary Figures S4A,B), suggesting that these were granule cells. The quantification of BARHL1^+^ cells by flow cytometry (Figure 3C) revealed a value of 46.2 ± 1.9% (Mean ± SEM of the three iPSC lines) on day 50, which is similar to the proportion of BARHL1^+^ cells found in 3D aggregates on day 35 (Figure 2K). The 2D cultures further showed distinct types of MAP2^+^ neurons with the nuclei stained for either TBR1 (Figure 3D and Supplementary Figure S4C), which identifies precursors of deep cerebellar nuclei projection neurons (Fink, 2006), or TBR2 (Figure 3E and Supplementary Figure S4D), which identifies precursors of unipolar brunch cells. Additional neurons expressed neurogranin in the cytoplasm (NRGN, Figures 3F,G and Supplementary Figures S4E,F) and PAX2 in the nucleus (Figure 3G and Supplementary Figure S4F), which are markers of Golgi cells (Singec et al., 2004; Leto and Rossi, 2012). Furthermore, immunostaining analysis using an antibody to calbindin (CALB), a calcium-binding protein highly expressed in Purkinje cells, revealed multiple positive cells (Figure 3H). In other neurons, immunostaining did not detect CALB but these cells were recognized by anti-parvalbumin antibodies (PVALB, Figure 3H and Supplementary Figure S4G, red staining), indicating that they were likely the precursors of GABAergic interneurons. Consistent with these immunofluorescence results, significant levels of mRNAs encoding the transcription factors PAX2, BARHL1, TBR1, and TBR2, as well as hallmark proteins of differentiated neurons, including the calmodulin-binding protein NRGN and the calcium-binding protein PVALB were detected by qRT-PCR analysis on days 56 and 80 (Figure 3I). Additionally, mRNAs encoding the GABAergic marker GAD65 and the glutamatergic marker VGLUT1 were significantly increased by day 80 of differentiation (Supplementary Figure S4H).

**FIGURE 4:**
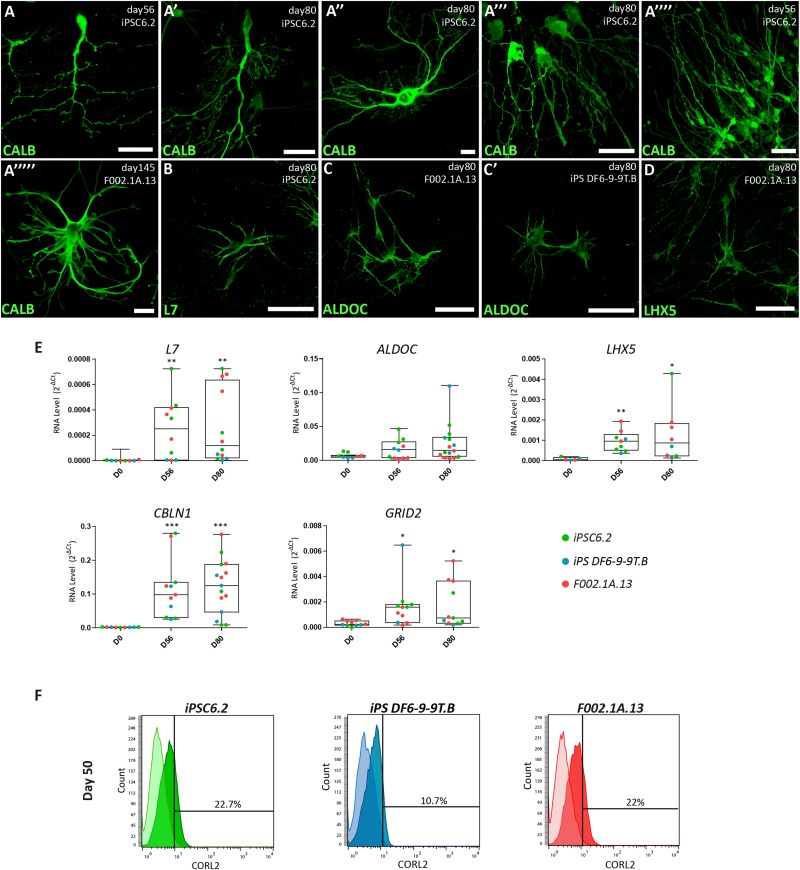
Characterization of Purkinje cells in 2D culture. **(A–D)** Immunofluorescence analysis on days 56, 80, and 145 of differentiation of the indicated iPSC lines using specific markers for Purkinje cells. Scale bars, 50 μm. **(E)** qRT-PCR analysis of cultures derived from each iPSC line at the indicated time-points. Box-plot diagrams depict mRNA expression levels (2^–Δ^ ^*Ct*^) relative to GAPDH. Each color-coded dot represents data from one differentiation experiment. One-way ANOVA (Dunn’s Multiple Comparison Test), ^∗^*p* < 0.05, ^∗∗^*p* < 0.01, ^∗∗∗^*p* < 0.001; error bars represent SEM. **(F)** Flow cytometry analysis of CORL2^+^ cells on day 50 of differentiation of the indicated iPSC lines.

Immunofluorescence analysis further revealed that 2D cultures derived from the three iPSC lines differentiated for 56, 80, or 145 days had neurons expressing markers of Purkinje cells, including the calcium-binding protein calbindin (CALB, Figures 4A–A””’), the Purkinje cell protein 2 (PCP2/L7, Figure 4B), aldolase C (ALDOC, also known as “zebrin II”, Figures 4C–C”), a brain type isozyme of a glycolysis enzyme, and the LIM-homeodomain transcription factor LHX5 (Figure 4D and Supplementary Figure S5). In agreement with these results, significant levels of mRNA encoding L7, ALDOC and LHX5 were detected by qRT-PCR analysis of cultures derived from the three iPSC lines differentiated for 56 and 80 days (Figure 4E). Additionally, we detected expression of mRNAs encoding the Purkinje cell-specific glutamate receptor GRID2, and the glycoprotein CBLN1 that controls synaptic plasticity and synapse integrity of Purkinje cells (Figure 4E). To determine the proportion of Purkinje cells in 2D cultures, flow cytometry was used to quantify CORL2^+^ cells. The results show that depending on the iPSC line, the percentage of post-mitotic Purkinje cells on day 50 ranged around 10 and 20% (Figure 4F).

### Assessing the Maturity of Cerebellar Neurons in 2D Culture

In order to assess the maturation of the cerebellar neurons, we next performed SCCI. Cells were preloaded with the calcium indicator fluorescent dye Fura-2 that switches its excitation peak from 340 to 380 nm in response to calcium binding, allowing the concentration of intracellular calcium to be determined based on the ratio of fluorescence emission after sequential excitation at 340 and 380 nm (Grienberger and Konnerth, 2012). Cells were stimulated by exposure to KCl at different time-points. If cells were differentiated into excitable neurons, high KCl concentrations were expected to induce the opening of voltage sensitive calcium channels resulting in massive influx of calcium into the cytoplasm (Ambrósio et al., 2000; Macías et al., 2001). Elevations in cytosolic calcium concentration (visualized by increased fluorescence ratios) were indeed observed in cells cultured for 50 days (Supplementary Figures S6A,B). These results confirm that our iPSC-derived cultures produced excitable neurons.

**FIGURE 5:**
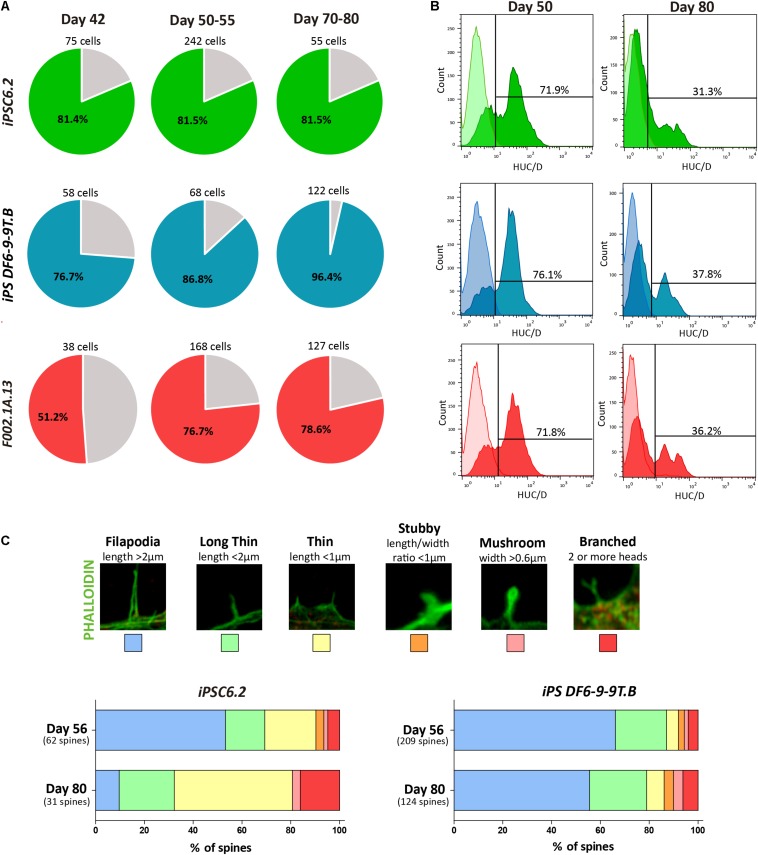
Assessment of neuronal maturation. **(A)** The percentage of responsive cells displaying a Histamine/KCl ratio below 0.8 is shown in colored slices. For each iPSC line three independent differentiation experiments were performed. **(B)** Flow cytometry analysis of HuC/D^+^ cells on days 50 and 80 of differentiation of the indicated iPSC lines. **(C)** Representative images of dendritic spine morphologies. Bars depict the relative proportion of the distinct dendritic spine morphologies observed in cultures derived from the indicated iPSC lines on days 56 and 80.

In contrast to differentiated neurons, stem cells and neuronal progenitors express functional histamine receptors (Agasse et al., 2008; Molina-Hernández and Velasco, 2008; Rodríguez-Martínez et al., 2012). KCl depolarization causes an increase in calcium concentration in neurons, whereas stimulation with histamine leads to an increase in calcium concentration in stem/progenitor cells (Agasse et al., 2008; Rodrigues et al., 2017). We therefore measured variations in intracellular free calcium concentration following 50 mM KCl and 100 μM histamine stimulation to discriminate between progenitors and differentiated neurons in our cultures (Supplementary Figure S6C). Histamine/KCl ratios were calculated using the corresponding peak values given by the normalized ratios of fluorescence at 340/380 nm. Neurons typically depict ratios below 0.8 whereas progenitor cells have ratios between 1 and 1.3 (Agasse et al., 2008; Rodrigues et al., 2017). Quantification of the percentage of cells displaying a Histamine/KCl ratio below 0.8 showed that on day 42 of differentiation, approximately 80% of cells in iPS-DF6-9-9T.B and iPSC6.2-derived cultures exhibited properties of differentiated neurons while in the F002.1A.13-derived culture approximately half of the cells were still progenitors (Figure 5A). Remarkably, by day 80 the vast majority of cells in either culture behaved as differentiated neurons (Figure 5A), indicating that neuronal differentiation was a gradual and continuous time-dependent process in our 2D cultures.

Consistent with the results from SCCI analysis, immunofluorescence on day 50 revealed cells staining for PAX6 but not expressing MAP2 or the astrocyte marker glial fibrillary acidic protein (GFAP), revealing the presence of progenitor cells in these cultures (Supplementary Figures S6D,E). In contrast, on day 80, the cultures consisted predominantly of a dense MAP2^+^ neuronal network with some scattered GFAP^+^ astrocytes (Supplementary Figures S6F,G). This finding suggests that by day 80 the cultures were mainly composed of differentiated neurons and glial cells. Moreover, we used flow cytometry to quantify the proportion of cells expressing HuC/HuD (HuC/D), which are RNA-binding proteins expressed specifically in newborn neurons (Abranches et al., 2009). As expected, the proportion of HuC/D^+^ newborn neurons in 2D cultures decreased from 72–76% on day 50 to 31–38% on day 80 (Figure 5B).

Because the morphology of dendritic spines changes during neuronal maturation (Hering and Sheng, 2001; Risher et al., 2014), we measured spine head width and neck length as shown in Figure 5C. Dendritic spines were classified as follows: Filopodia (length > 2 μm); Long thin (length < 2 μm); Thin (length < 1 μm); Stubby (length/width ratio < 1 μm); Mushroom (width > 0.6 μm), and Branched (2 or more heads), as previously described (Risher et al., 2014). As shown in Figure 5C, from day 56 to day 81, the relative proportion of more mature branched spines increased, further confirming the progressive maturation of neurons over time in 2D culture.

**FIGURE 6:**
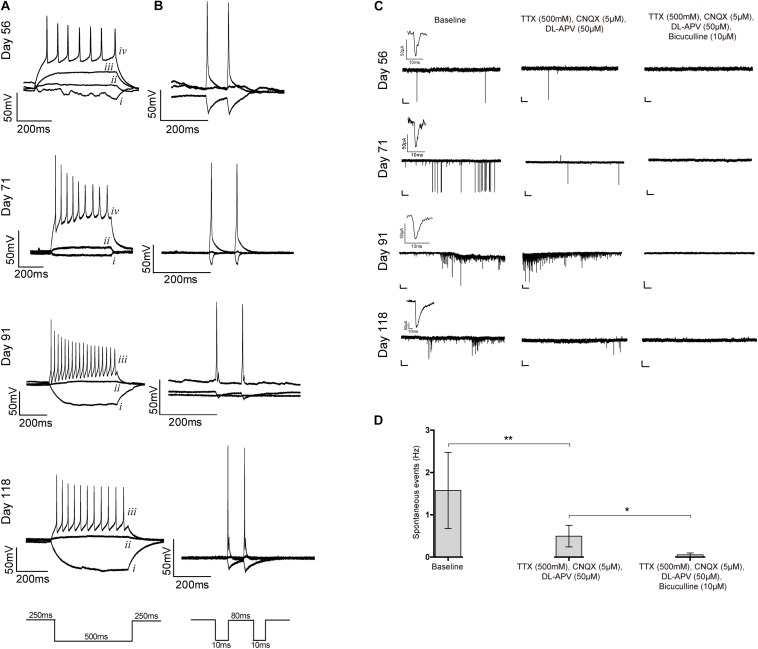
Patch-clamp recordings. **(A–C)** Whole-cell patch clamp recordings for iPSC6.2-derived neurons on the indicated days of differentiation. **(A)** Representative traces of firing responses evoked by 500 ms current pulses. Each trace corresponds to a single injection current of –25 pA (*i*), 0 pA (*ii*), +25 pA (*iii*) or +50 pA (*iv*). **(B)** Firing responses to two independent current injections (10 ms) separated by 80 ms. **(C)** Representative traces of spontaneous postsynaptic currents recorded without any treatment (left), after blocking voltage dependent sodium channels using TTX and ionotropic glutamate receptors with CNQX and DL-APV (middle), and GABA_A_ receptors with bicuculline (right); an example of a miniature postsynaptic current is also shown in each case. Recordings in each row are from the same cell on the indicated days in culture. Scale bars correspond to 50 pA and 2000 ms. **(D)** Frequency of spontaneous events recorded without any treatment (baseline) and after addition of the indicated blockers. Data from three independent differentiation experiments using iPCS6.2-derived cells. Student’s *t*-test (two-tailed) statistics, **p < 0.05, **p < 0.01; error bars represent SEM.

Finally, we evaluated the electrophysiological properties of differentiated cells by using patch-clamp recordings. Cells analyzed on days 56 through 118 presented typical neuronal fire action potentials upon a current injection and were also able to depolarize, repolarize and recover, responding to a second current injection (Figures 6A,B). Most cells analyzed after 80 days of differentiation were spiking and showed high amplitude action potential (indicative of expression of voltage-dependent Na^+^ channels) and reduced spike width (indicative of abundant K^+^ channels), as expected for differentiated neurons. Furthermore, spontaneous currents were recorded, which indicates the presence of synaptic connections (Figure 6C and Supplementary Figure S7A). A subset of these currents remained in the presence of the sodium channel blocker TTX (indicative that they are independent of action potential generation) and of specific antagonists of ionotropic glutamate receptors, CNQX and DL-APV, suggestive of the existence of functional GABAergic synapses. Addition of the GABA_A_ receptor antagonist bicuculline completely abolished all activity (Figures 6C,D), confirming that the previously recorded miniature events indeed resulted from the spontaneous activity of GABAergic synapses. To confirm these spontaneous inhibitory and excitatory outputs, GABAergic activity was first inhibited by using bicuculline (Supplementary Figure S7B). Indeed, a subset of spontaneous currents were maintained, suggesting the presence of Glutamatergic activity (Supplementary Figure S7B). Addition of CNQX and DL-APV completely blocked the synaptic activity (Supplementary Figure S7B), which confirmed the existence of Glutamatergic synapses. These data show first, that neurons in 2D culture established functional connections and second, that an active GABAergic and Glutamatergic neuronal network was created.

## Discussion

Using only a defined medium and without the need for co-culturing the cells, we successfully generated reproducible network-forming GABAergic and Glutamatergic cerebellar neurons derived from three distinct human iPSC lines.

We started with a 3D culture system and we controlled the sizes of cell aggregates as previously described (Ungrin et al., 2008), leading to an efficient neuronal commitment (Bauwens et al., 2008; Miranda et al., 2015). We produced hundreds of cell aggregates per cm^2^, which is a significant increase over the 96-well plates used by most cerebellar differentiation protocols previously described (Ungrin et al., 2008; Bratt-Leal et al., 2009; Muguruma et al., 2015; Wang et al., 2015). We cultured cells in chemically defined medium with sequential addition of FGF2, SB, FGF19, and SDF1 to induce the spontaneous formation of a cerebellar plate neuroepithelium, which differentiated into a multilayered structure reminiscent of cerebellar ontogenesis *in vivo* (Muguruma et al., 2015; Ishida et al., 2016). The initial stage of cerebellar commitment in 3D aggregates was detected on day 7, when expression of *FGF8* mRNA was at its highest and dropped thereafter. This *in vitro* behavior recapitulates cerebellar ontogenesis *in vivo* (reviewed in Marzban et al., 2015). Indeed, previous *in vivo* studies have shown that at early embryonic stages FGF signaling is required to establish the cerebellar territory, however, its suppression afterward is essential for cerebellar development (Suzuki-Hirano et al., 2010; Butts et al., 2014).

In our study, cell aggregates formed neural tube-like structures organized in layers with apico-basal polarity. Most important, compared to findings from a previous study (Muguruma et al., 2015), the aggregates in our cultures exhibited an earlier neuronal differentiation pattern. On day 35, cells were already positive for OLIG2, CORL2, and BARHL1. OLIG2 is the earliest marker for Purkinje cells, and is associated with cell cycle exit and differentiation into post-mitotic neurons (Takebayashi et al., 2002; Seto et al., 2014; Ju et al., 2016). CORL2 is specifically expressed in post-mitotic Purkinje cell precursors shortly after exiting the cell cycle (Minaki et al., 2008) and plays an essential role in Purkinje cell development (Wang et al., 2011; Nakatani et al., 2014). In contrast, BARHL1 is expressed in migrating granule cell precursors (Bulfone et al., 2000). These results indicate that Purkinje and granule cell precursors formed in our iPSC-derived cultures by day 35. Our cultures further had cells positive for GAD65, the protein glutamic acid decarboxylase isoform 65, which is a rate-limiting GABA synthesizing enzyme localized primarily on presynaptic boutons (Esclapez et al., 1994). Because the onset of GAD65 expression in the cerebellum occurs after synaptogenesis (Greif et al., 1991), we conclude that an interconnected neuronal network formed in our cultures by day 35.

Our differentiation strategy replaces co-culture by a defined basal medium optimized for neuronal cell culture (Bardy et al., 2015). Upon dissociation of cell aggregates on day 35 and re-plating on a laminin-coated surface, cerebellar precursors differentiated without the need for co-culturing. Remarkably, our 2D cultures remained viable for up to 145 days. On day 50, the percentage of post-mitotic Purkinje precursors (CORL2^+^) in the 2D cultures ranged around 10 and 20%, depending on the iPSC line. The presence of cells expressing calbindin, a calcium-binding protein highly abundant in Purkinje cells (Nag and Wadhwa, 1999; Whitney et al., 2008), as well as additional late markers of Purkinje cells, including the Purkinje cell protein 2 (PCP2/L7, Oberdick et al., 1988; Zhang et al., 2002), the glycolysis enzyme aldolase C (ALDOC, Royds et al., 1987), the transcription factor LHX5 (Zhao et al., 2007), and the Purkinje cell-specific glutamate receptor GRID2 (Araki et al., 1993), strongly indicates that our culture conditions support the development of Purkinje cells by day 80 and onward.

Furthermore, our cultures had MAP2^+^ neurons with distinctive morphology that stained for PAX6 in the nucleus, suggesting that these were granule cells. Another subset of neurons in our cultures were negative for calbindin but expressed parvalbumin, suggesting that they were GABAergic interneurons (Bastianelli, 2003). Others showed a strong positive signal for neurogranin and PAX2, which is highly expressed in Golgi cells in the mouse cerebellum (Singec et al., 2004; Leto and Rossi, 2012). Furthermore, MAP2^+^ neurons expressing either TBR1 or TBR2 in the nucleus revealed the formation of deep cerebellar nuclei projection neurons (Fink, 2006) and unipolar brush cells (Englund, 2006), respectively.

Taken together, the results obtained with SCCI and patch-clamp recordings demonstrate that our culture conditions support the differentiation of iPSCs into electrophysiologically active cerebellar neurons. In particular, the presence of synaptic connections shows that a functional neuronal network has been generated.

Although we observed a progressive maturation of neurons over time in 2D culture, our cells did not reach the level of neuronal maturation equivalent to that occurring in post-natal human cerebellum. This is particularly evident for Purkinje cells, which in our cultures did not form the elaborated dendritic branches observed *in vivo*. Further studies should search for additional signaling molecules that are necessary, for *in vitro* differentiated cells to fully recapitulate the maturation processes that occur in the developing human cerebellum.

## Conclusion

Here we show for the first time that is possible to generate different types of electrophysiologically active GABAergic and glutamatergic cerebellar neurons in long-term cultures without co-culturing with other cell types. Our findings represent an important contribution toward the development of autologous replacement strategies for the treatment of cerebellar degenerative diseases. Additionally, functional cerebellar neurons are an important cell source for drug screening and for the study of specific pathways involved with the development of cerebellar diseases such as ataxias, a group of disorders that affect many children and adults worldwide.

## Data Availability Statement

All datasets generated for this study are included in the article/Supplementary Material.

## Author Contributions

All authors contributed to design research. TS performed the culture cell differentiations and cell culture characterization. EB, TF, and CR assisted with cell culture maintenance and growth. TF assisted with flow cytometry experiments. EB and TF contributed with data analysis. TS and SV performed the single cell calcium imaging and patch clamp experiments. TS and MC-F wrote the manuscript.

## Conflict of Interest

The authors declare that the research was conducted in the absence of any commercial or financial relationships that could be construed as a potential conflict of interest.
