# Supplementary material for: Maturation of Human Pluripotent Stem Cell-Derived Cerebellar Neurons in the Absence of Co-culture
Source: Front Bioeng Biotechnol. 2020 Feb 14;8:70. doi: 10.3389/fbioe.2020.00070 (PMC7033648; doi:10.3389/fbioe.2020.00070)
Supplement: Supplementary file 1 [file Data_Sheet_1.PDF]

Supplementary Material

Supplementary Figures

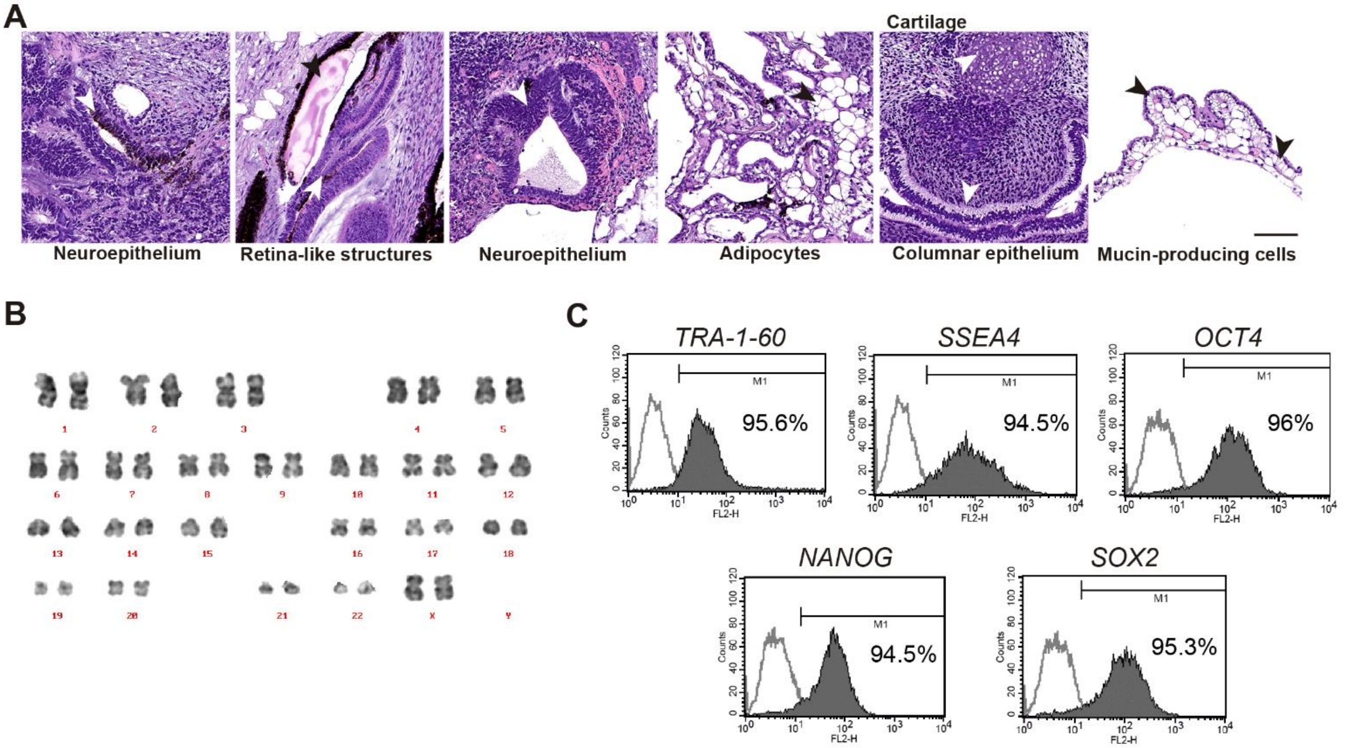

Supplementary Figure S1. Teratomas formed after subcutaneous injection of F002.1A.13 cells. (A) Representative histological sections of teratomas stained with hematoxylin and eosin. Tumors contained cell types derived from the three embryonic germ layers, including ectodermal components corresponding to neuroepithelium with melanin pigment, retina-like pigmented structures, and neuroepithelium consisting of small hyperchromatic cells arranged in rosettes; mesodermal components corresponding to adipocytes and cartilage; and endodermal components corresponding to columnar epithelium and mucin-producing cells, as indicated by arrowheads in each panel. **(B)** Karyotype performed by Giemsa Trypsin banding. **(C)** Flow cytometry analysis for the indicated pluripotency markers on day zero of differentiation.

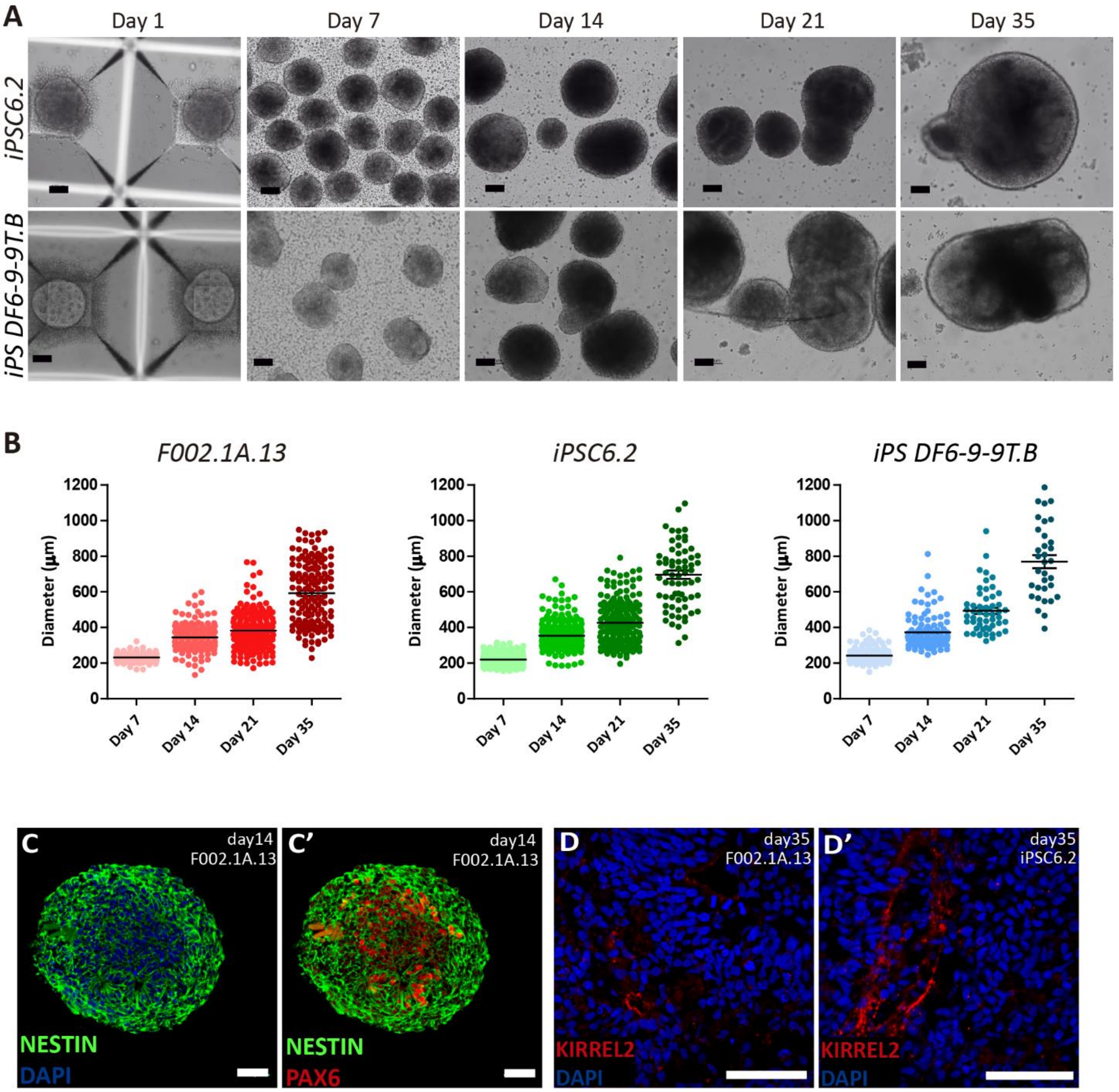

Supplementary Figure S2. Characterization of cerebellar progenitors differentiated in 3D culture. **(A)** The indicated human iPSC lines were induced to aggregate and develop into 3D structures. Photos were taken at the indicated time points. Scale bar, 100 µm. **(B)** The diameter of floating aggregates obtained from different iPSC lines was estimated at the indicated time points. Results from 3 independent experiments. **(C, C’)** Immunofluorescence for PAX6 and NESTIN in aggregates derived from F002.1A.13 cells on day 14 of differentiation (scale bar, 50µm). **(D, D’)** Immunofluorescence for KIRREL2 in F002.1A.13 and iPSC6.2-derived aggregates on day 35 of differentiation (scale bar, 50µm).

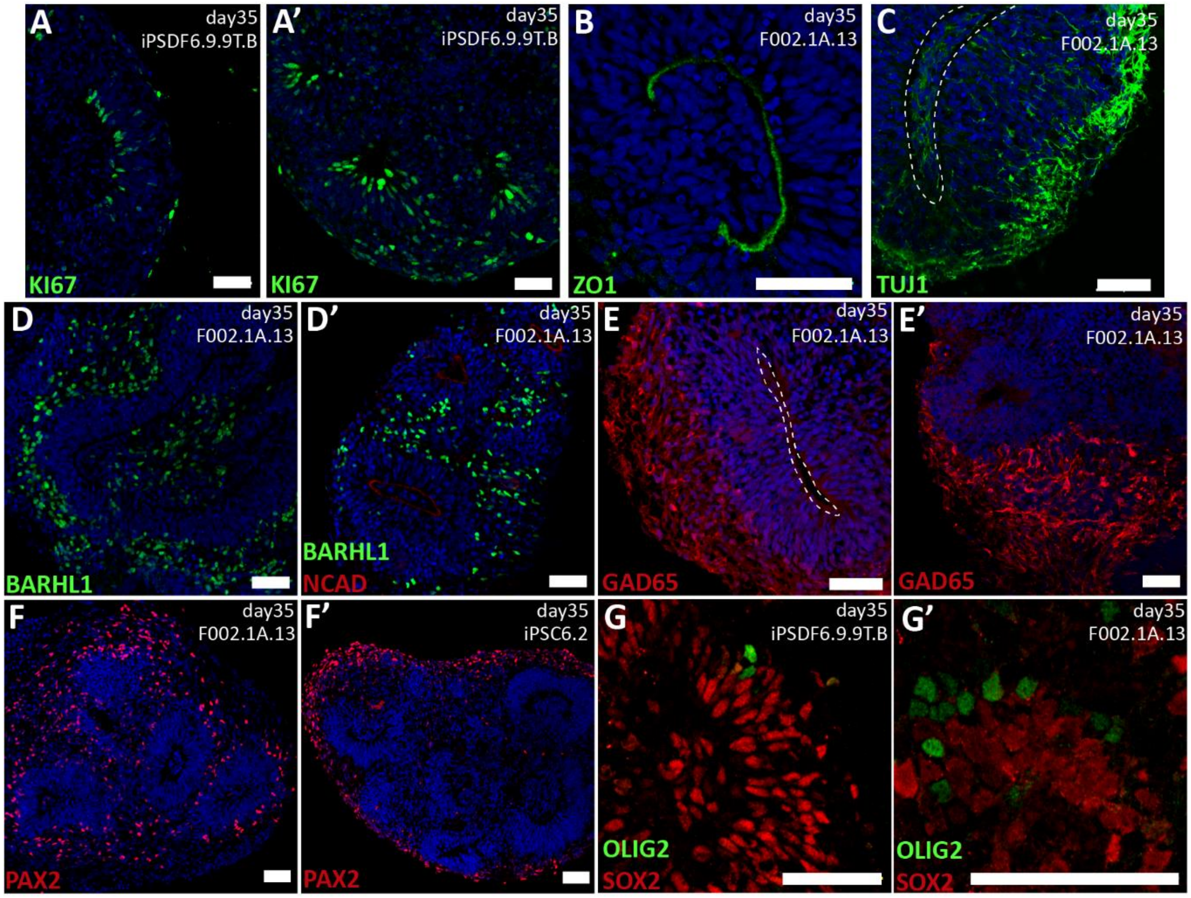

Supplementary Figure S3. Characterization of neuro-epithelial rosettes. (A-G) Immunofluorescence analysis on day 35 of differentiation of the indicated iPSC lines. Blue staining corresponds to nuclei labeled with DAPI. Dashed line delineates the lumen. Scale bars, 50μm.

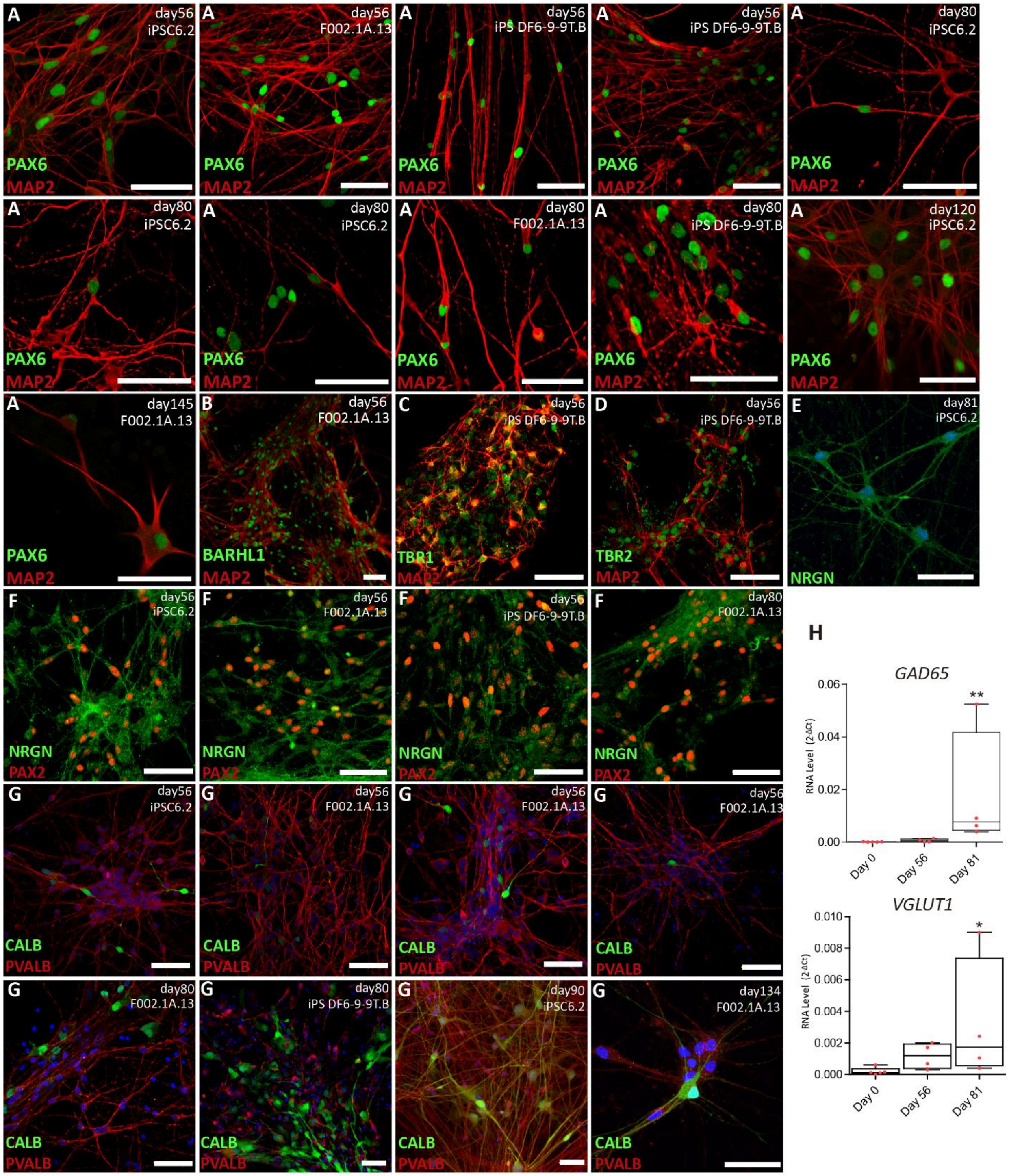

Supplementary Figure S4. Characterization of cerebellar neurons in 2D culture. (A-G) Immunofluorescence for the indicated proteins in neurons derived from different iPSC lines. Scale bars, 50µm. **(H)** qRT-PCR analysis of cultures derived from F002.1A.13 cells at the indicated time points. The graphs depict mRNA expression levels (2-ΔCt) relative to GAPDH. Each dot represents data from an independent experiment (n=4). One-way ANOVA (Dunn's Multiple Comparison Test), * $p<0.05$, ** $p<0.01$; error bars represent SEM.

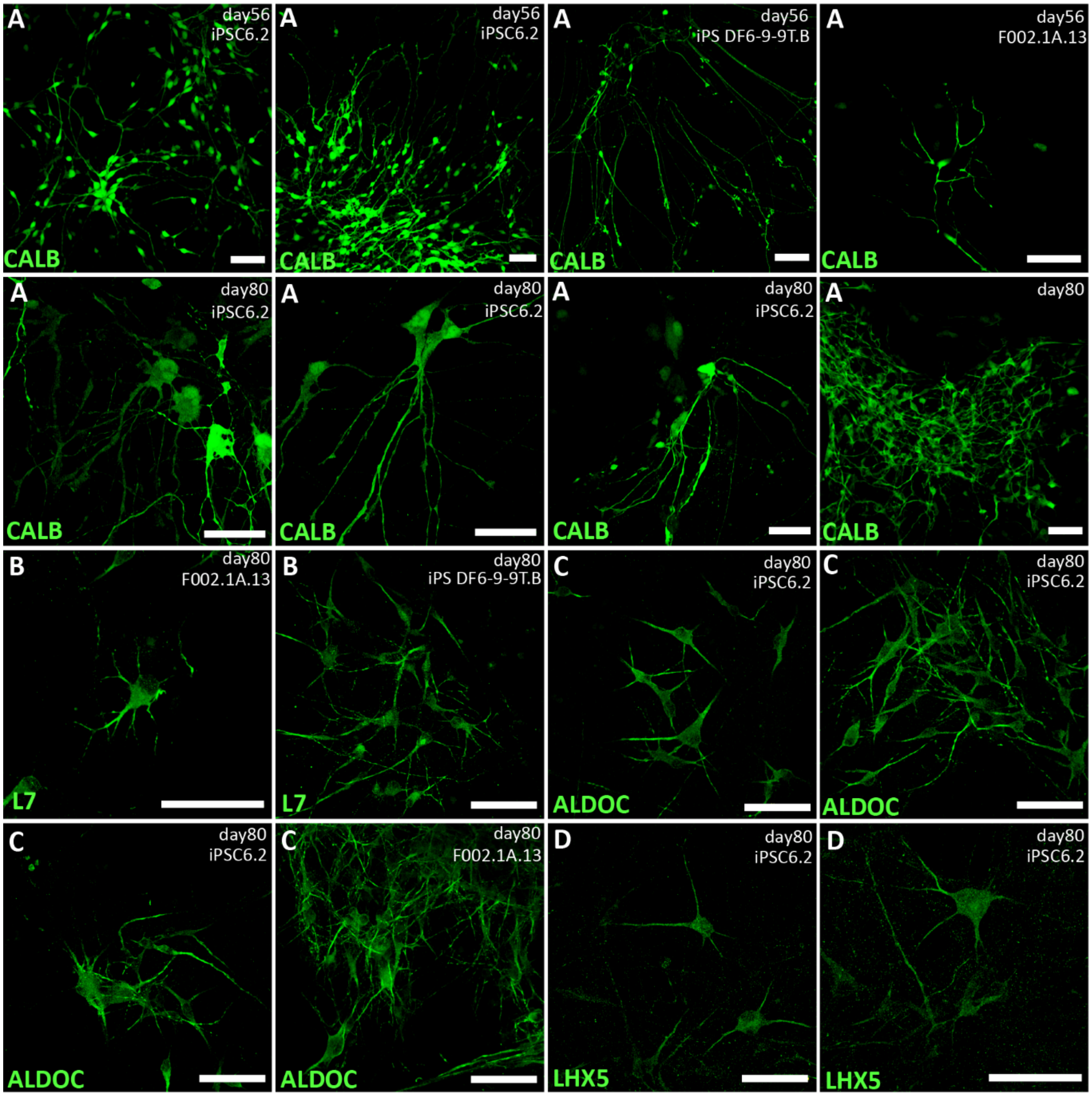

Supplementary Figure S5. Characterization of Purkinje cells in 2D culture. (A-D) Immunofluorescence analysis on days 56 and 80 of differentiation of the indicated iPSC lines using specific markers for Purkinje cells. Scale bars, 50µm.

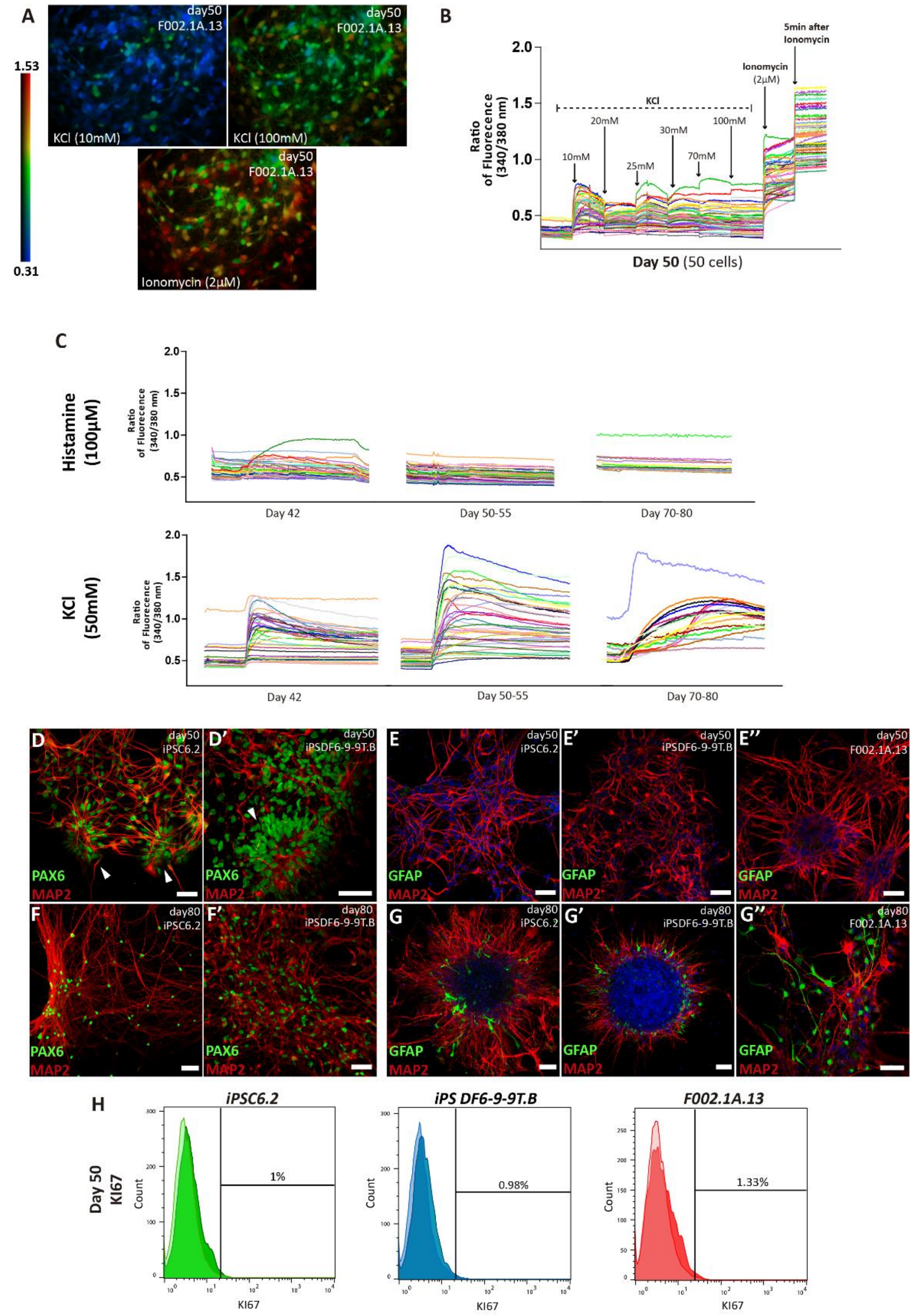

Supplementary Figure S6. Assessment of neuronal maturation. (A) Representative ratio images (left) and fluorescence ratio profiles (right) of individual F002.1A.13-derived cells on day 50. Images were taken immediately after cells received the indicated stimulus. **(B)** Representative fluorescence ratio profiles after stimulation of cells in iPCS6.2-derived culture. **(D-G)** Immunofluorescence analysis using the indicated markers. Scale bars, 50µm. **(H)** Flow cytometry analysis of $KI67^+$ cells on day 50.

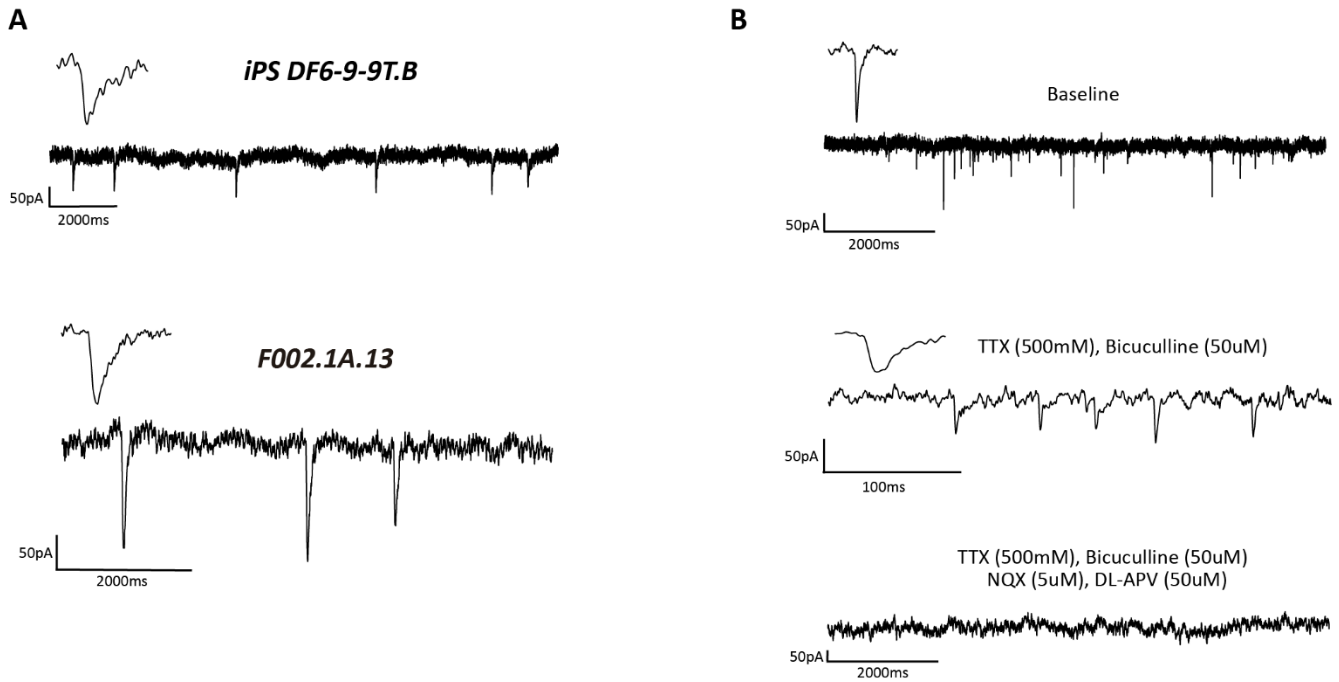

Supplementary Figure S7. Patch-clamp recordings. (A) Representative traces of spontaneous postsynaptic currents recorded without any treatment for DF6.9.9T.B and F002.1A.13-derived neurons on day 56 of differentiation. **(B)** Representative traces of spontaneous postsynaptic currents recorded without any treatment, after blocking voltage dependent sodium channels using TTX and $GABA_A$ receptors with bicuculline, and ionotropic glutamate receptors with CNQX and DL-APV; an example of a miniature postsynaptic current is also shown in each case. Recordings are from iPSC6.2-derived neurons at day 80.

Supplementary Tables

Supplementary Table S1. Primary antibodies and dilutions used for Immunostaining

Antibody	Supplier company	Host species	Dilution
BARHL1	Atlas	rabbit	1:500
ALDOC	Santa Cruz Biotechnology	mouse	1:200
CALBINDIN	Swant	rabbit	1:500

CORL2	Atlas	rabbit	1:100
EN2	Sigma	mouse	1:200
KI67	Abcam	rabbit	1:100
KIRREL2	R&D Systems	mouse	1:100
L7/PCP2	Santa Cruz Biotechnology	mouse	1:200
LHX5	Santa Cruz Biotechnology	mouse	1:200
MAP2	Sigma	mouse	1:1000
N-CADHERIN	BD Transduction	mouse	1:1000
NESTIN	R&D	mouse	1:400
NEUROGRANIN	Millipore	rabbit	1:200
OLIG2	Millipore	rabbit	1:500
PARVALBUMIN	Sigma	mouse	1:200
PAX2	Abnova	mouse	1:400
PAX6	Covance	rabbit	1:400
SOX2	R&D	mouse	1:200
TBR1	Millipore	rabbit	1:200
TBR2	Abcam	rabbit	1:200
TUJ1	Biolegend	mouse	1:1000
ZO-1	Novex	rabbit	1:100

Supplementary Table S2. Primers used for qRT-PCR

Gene	Forward Primer	Reverse Primer
ALDOC	ACTCCATACCACAGCCCTTG	GCAATTTCTTCTGCCCTCAG
ATOH1	TGTTATCCCGTCGTTCAACAAC	TGGGCGTTTGTAGCAGCTC
BARHL1	GAGCGGCAGAAGTACCTGAG	GTAGAAATAAGGCGACGGGAAC
CBLN1	TTTGATTCAGAACGCAGCAC	TTGGATTAGGACTCCGTTGC
CORL2	CCAGGTGTTAAAAGGAAACACA	GCTCCCTTTTCATCTGATCCT
EN2	CCGGCGTGGGTCTACTGTA	GGCCGCTTGTCCTCTTTGTT
FGF8	GAGCCTGGTGACGGATCAG	CGTTGCTCTTGGCGATCAG
GAD65	GTCTCCAGCTCGCATACACA	CGAAAGACCAAAAGCCAGAG
GAPDH	GAGTCAACGGATTTGGTCGT	TTGATTTTGGAGGGATCTCG
GRID2	AGCTCTTCCTCTCTTGGTTTCC	GCCCCACGTTGCCTAGAAAT
L7/PCP2	ACCAGGAGGGCTTCTTCAAT	CTGTCACACGTTGGTCATCC
LHX5	CAGCAACGCTGTAGCCAATTT	TCCGGATCCTCATCTTTGTC
NESTIN	GAAACAGCCATAGAGGGCAAA	TGGTTTTCCAGAGTCTTCAGTGA
NEUROGRANIN	TCAAAGTTCCCGAGGAGAGA	CTAAAAGGGCACGGACTCAG
OLIG2	GACAAGCTAGGAGGCAGTGG	CGGCTCTGTCATTTGCTTCTTG
OTX2	AGAGGACGACGTTCACTCG	TCGGGCAAGTTGATTTTCAGT
PARVALBUMIN	TTCTCCCCAGATGCCAGAGA	GAGATTGGGTGTTCAGGGCA
PAX2	AACGACAGAACCCGACTATGT	GAGCGAGGAATCCCCAGGA
TBR1	CGTCTGCAGCGAATAAGTGC	AATGTGGAGGCCGAGACTTG
TBR2	CACATTGTAGTGGGCAGTGG	CGCCACCAAACTGAGATGAT
VGLUT1	TACACGGCTCCTTTTTCTGG	CTGAGGGGATCAGCATGTTT

Supplementary Table S3. Primary antibodies and dilutions used for Flow cytometry

Antibody	Supplier company	Host species	Dilution
BARHL1	Atlas	rabbit	1:500
CORL2	Atlas	rabbit	1:100
HuC/D	ThermoFisher	mouse	1:200
KI67	BD Biosciences	mouse	1:20
NANOG	Millipore	rabbit	1:300

OCT4	ThermoFisher	mouse	1:300
SEEA-4-PE	Miltenyi Biotec	mouse	1:10
SOX2	R&D	mouse	1:200
Tra-1-60-PE	Miltenyi Biotec	mouse	1:50
